# Association and Risk Factors for Hypertension and Dyslipidemia in Young Adults from Poland

**DOI:** 10.3390/ijerph20020982

**Published:** 2023-01-05

**Authors:** Justyna Wyszyńska, Edyta Łuszczki, Grzegorz Sobek, Artur Mazur, Katarzyna Dereń

**Affiliations:** 1 Institute of Health Sciences, Medical College of Rzeszow University, 35-959 Rzeszow, Poland; 2 Institute of Medical Sciences, Medical College of Rzeszow University, 35-959 Rzeszow, Poland

**Keywords:** dyslipidemia, hypertension, risk factors, young adults

## Abstract

Hypertension and dyslipidemia are major risk factors for cardiovascular disease. Studies on the association between abnormal levels of lipids and hypertension have yielded inconsistent results. The aim of this study was to examine the prevalence of hypertension and dyslipidemia and its risk factors in young Polish adults. Furthermore, the association between plasma lipid levels and the risk of hypertension was determined. A cross-sectional study was conducted among 115 volunteer participants. Blood pressure was measured using an automated oscillometric sphygmomanometer. Blood lipids were analyzed from a fasting blood sample received by finger prick. Body fat percentage was assessed using a bioelectrical impedance analysis device. Socioeconomic and lifestyle factors (age, date of birth, place of residence, screen time, and tobacco use) were self-reported by the participant. The prevalence of hypertension was higher in men than in women (61.5 vs. 21.3%). The prevalence of elevated TC, TG, high LDL, and low HDL was 22.6%, 7.8%, 38.3%, and 13.9%, respectively. Spending more than 2 h daily in front of a computer was identified as a significant predictor of hypertension and elevated TG levels (*p* < 0.05). A high number of cigarettes smoked daily was a significant risk factor for hypertension (*p* = 0.047). Hypertension contributed to a higher risk of abnormal values of TC (OR = 5.89), LDL (OR = 5.38), and TG (OR = 9.75). Participants with hypertension were more likely than normotensive subjects to have elevated levels of TC, LDL, and TG. The prevalence of hypertension was significantly higher in young men than in women. BMI was associated with the prevalence of hypertension and elevated TC levels. Spending more than 2 h per day in front of a computer contributed to the prevalence of hypertension and elevated TG levels. Participants with hypertension smoked a higher number of cigarettes daily compared to those with normotension.

## 1. Introduction

Hypertension is a chronic disease that is highly prevalent in the adult population of Poland and throughout the world, especially among people over 60 years of age. It is the most significant risk factor for cardiovascular disease including coronary artery disease, stroke, myocardial infarction, or heart failure [1]. According to the World Health Organization (WHO), elevated blood pressure (BP) is the leading risk factor for death in the world [2]. According to American epidemiological studies, hypertension is responsible for up to 40.6% of cardiovascular mortality, while smoking is responsible for 13.2%, unhealthy diet for 11.9%, and insufficient physical activity levels for 8.8% [3]. Modifiable risk factors include unhealthy diets (excessive salt consumption, a diet high in saturated fat and trans fats, and low intake of fruits and vegetables), low levels of physical activity, consumption of tobacco and alcohol, and being overweight or obese [4].

Dyslipidemia, also one of the main risk factors for cardiovascular diseases, is one or a combination of elevated total cholesterol (TC), high low-density lipoprotein (LDL), low high-density lipoprotein (HDL), and elevated triglyceride (TG) [1]. Approximately 80% of lipid disorders are associated with diet and lifestyle [5]. Modifiable risk factors, including a diet high in saturated or trans fats, sedentary lifestyle, smoking, and excess body weight, increase the risk of dyslipidemia [6]. The prevalence of dyslipidemia is much higher among patients with co-existing cardiovascular risk factors such as hypertension, diabetes, or the human immunodeficiency virus [7].

Dyslipidemia could be associated with hypertension by several mechanisms. Atherosclerosis resulting from lipid abnormalities can cause structural changes in large arteries, resulting in a reduction in elasticity [8]. Furthermore, endothelial dysfunction due to dyslipidemia that results in reduced nitric oxide production, release, and activity, as well as abnormal vasomotor activity, could manifest as hypertension [9]. Furthermore, lipid-mediated impairment of the renal microvasculature could manifest as hypertension [10].

To date, few studies have examined whether lipid levels are associated with the risk of hypertension in young adults [11,12,13]. Data on the relationship between hypertension and lipid profile among the young Polish population are rare in the literature and inconclusive. Recognizing risk factors for hypertension and dyslipidemia is crucial to developing effective intervention programs for the prevention of these diseases. Therefore, using data from a cross-sectional study of young adults, we examined the prevalence of hypertension and dyslipidemia and its risk factors in Poland. Moreover, we determined the association between plasma lipids levels and the risk of hypertension.

## 2. Materials and Methods

### 2.1. Study Design and Population

Written informed consent was obtained from the participants prior to participation in the study. The study was approved by the Bioethics Committee at the Medical Department of the University of Rzeszów, Poland (No. 2018/06/11), and it was conducted in accordance with ethical standards laid down in an appropriate version of the Declaration of Helsinki. The study was conducted from October to November 2020.

A cross-sectional study was conducted among volunteer participants from high schools in Rzeszów. Three hundred students were invited to participate in a health survey. Forty three percent (*n* = 129) of those invited attended. The attendance rate was higher in women than in men (99 women, 30 men). The following eligibility criteria were used for inclusion in the study: consent of the participant for participation in the study, an age of ≥18 years, and health status allowing for the examinations to be carried out. After the exclusion of 14 participants with missing values for any of these measurements, 89 women and 26 men were included in the present analysis.

### 2.2. Measurements

#### 2.2.1. Anthropometric Measurements

The body weight and height of the participants were measured using the standard protocol and equipment that was calibrated before and during the data collection period. Body height was measured upright, barefoot, to the nearest 0.1 cm using a portable stadiometer (HR-200, Tanita). Body mass was assessed with an accuracy of 0.01 kg using a body composition analyzer (MC-980 MA, Tanita). Body mass index (BMI) was calculated as weight in kilograms divided by height in meters squared (kg/m^2^).

#### 2.2.2. Blood Pressure

BP was measured using the current protocol guidelines for BP measurement after the participant had rested for 10 min [14]. Three measurements were taken with an automated oscillometric sphygmomanometer, Welch Allyn Inc., 4200B-E2 (Skaneateles Falls, NY, USA) along with a set of cuffs of various widths. Hypertension was defined according to the 2017 Guideline for High Blood Pressure in adults, as systolic blood pressure (SBP) ≥ 140 mmHg and/or diastolic blood pressure (DBP) ≥ 90 mmHg, or treatment for hypertension [15].

#### 2.2.3. Lipids Level

Blood lipids were analyzed from a fasting blood sample received by finger prick. Participants were advised to fast for 10–12 h before the test. Blood was analyzed immediately using a Cholestech LDX Analyzer (Cholestech Corporation). The results obtained using this device were well correlated with the measures obtained by other means [16]. The device was calibrated each day prior to use. TC, HDL, LDL, and TG levels were determined. To define an abnormal level of lipid/lipoproteins, we used cut-off points suggested in the 2020 Guidelines of the Polish Society of Laboratory Diagnostics and the Polish Lipid Association on Laboratory Diagnostics of lipid metabolism disorders: TC (≥190 mg/dL), HDL (≤45 mg/dL in women; ≤40 mg/dL in men), LDL (≥115 mg/dL), and TG (≥150 mg/dL) [17].

#### 2.2.4. Body Fat Percentage

Body fat percentage was assessed using a bioelectrical impedance analysis (BIA) device (MC-980 MA, Tanita). The BIA is a reliable and accurate tool for the measurement of body fat in the adult population [18]. BIA was performed in the early morning after overnight fasting for at least 8 h, since food or beverage consumption may decrease impedance by 4–15 Ω over a 2–4 h period after meals, representing an error smaller than 3% [19].

#### 2.2.5. Socio-Demographic/Lifestyle Factors

Socioeconomic and lifestyle factors (age, date of birth, place of residence, screen time, and tobacco use) were self-reported by the participants.

### 2.3. Statistical Analysis

Statistical analysis was performed using SPSS 25 software (IBM, North Harbour, UK). Statistics are presented as mean (SD) and n (%). The independent *t*-test and the Mann-Whitney test were used to determine a statistically significant difference between boys and girls. The prevalence of blood pressure status and abnormal blood lipid values was calculated using the independence test χ^2^. The risk factors for hypertension were determined by the independence test of independence χ^2^, a one-way analysis of variance, the Kruskal-Wallis test, and the risk factors for the prevalence of lipid abnormalities were determined by the independence test of independence χ^2^, a *t*-test for independent samples, and the Mann-Whitney test. Moreover, odds ratio (OR) with a 95% confidence interval (CI) was calculated. The level of statistical significance was adopted at *p* < 0.05.

## 3. Results

The final sample consisted of 115 participants aged 20 to 23 years (mean age 20.9 years; 77.4% female). Significant differences in height, weight, BMI, body fat percentage, SBP, and weekend time spent sitting in front of a computer were found between girls and boys (*p* < 0.001). The mean SBP was 117.8 mm Hg and 134.3 mm Hg in women and men, respectively. A significantly higher body fat content was diagnosed in women compared to men (24.1 vs. 17.0%). There was no significant difference in TC, HDL, LDL, and TG levels in women and men (Table 1).

Table 2 shows the overall prevalence of blood pressure status in women and men. Hypertension was diagnosed in 30.5% of the study population with significantly higher frequency in men than in women (61.5% vs. 21.3%, respectively).

Table 3 presents the risk factors for hypertension in the study population. BMI is significantly higher in participants with hypertension compared to those with normotension (22.7 vs. 20.0 kg/m^2^). Participants in cities with over 100,000 inhabitants and those who spend a long time in front of a computer on weekends were more likely to have hypertension. Additionally, the number of cigarettes smoked daily was significantly higher in subjects with hypertension compared to those with normotension (10.0 vs. 2.5, respectively).

Supplementary Table S1 presents a post hoc analysis of the prevalence of blood pressure categories and places of residence. Significant differences in the prevalence of elevated blood pressure were found between inhabitants of rural areas (34.0%) and those living in cities with fewer than 100,000 inhabitants (36.0%) and residents of large cities with over 100,000 inhabitants (8.1%).

The prevalence of abnormal values of blood lipids is presented in Table 4. The most frequent lipid abnormalities in the entire study population were high levels of LDL (38.3%). Higher levels of TC were observed in 22.6%, low levels of HDL in 13.9%, and elevated TG in 7.8% of the study population. There was no significant difference in the prevalence of lipid abnormalities between men and women.

Table 5 presents the risk factors for abnormalities in levels of TC and HDL. Significantly higher BMI was observed in participants with abnormal TC values (by 1.4 kg/m^2^). 

Table 6 presents the risk factors for abnormalities in levels of LDL and TG. Elevated TG values were observed in participants who were in front of a computer for 2 h daily during the week (*p* = 0.027).

Table 7 presents the associations between lipid level and blood pressure status. TC, LDL, and TG levels were significantly higher in participants with hypertension than in those with normal blood pressure status, by 25.2, 36.5, and 32.8 mg/dL, respectively. HDL levels were significantly lower in participants with hypertension (56.9 vs. 64.6 mg/dL).

Table 8 presents odds ratios for the prevalence of hypertension according to blood lipid status. Participants with abnormal TC levels had a risk of hypertension that was almost six times higher than that of subjects with acceptable TC levels (OR = 5.89). Elevated levels of LDL and TG contributed to an increased risk of hypertension (OR = 5.38 and 9.75, respectively).

## 4. Discussion

We investigated the association between lipid profile and hypertension among a population of young adults from Poland. Our results show that TC, LDL, and TG levels were significantly higher (at 25.2, 36.5, and 32.8 mg/dL, respectively) in hypertensive participants than in participants with normal blood pressure. HDL levels were significantly lower in participants with hypertension (56.9 vs. 64.6 mg/dL). Hypertension is the leading direct cause of death worldwide and one of the most important risk factors for cardiovascular disease. Elevated blood pressure often co-occurs with lipid disorders and is an additional factor that increases cardiovascular risk [12]. Around 80% of people with hypertension have comorbidities such as obesity, glucose intolerance, and lipid metabolism disorders, among others [20]. Our study showed that hypertension was present in 30.5% of the study population with a significantly higher prevalence in men than in women. A study by Midha et al. showed that the prevalence of hypertension was 32.8% in the urban population and 14.5% in rural areas [21]. In urban areas, people with hypertension were less physically active and more likely to smoke and consume alcohol. Approximately 9.2% of hypertensive people had coexisting diabetes. The mean body weight, BMI, and waist circumference of hypertensive people were significantly higher [21].

Our own study showed that BMI was significantly higher in hypertensive participants compared to normotensive participants (22.7 vs. 20.0 kg/m^2^). Participants in urban cities and those who spent more than 2 h in front of a computer on weekends were more likely to be hypertensive. Additionally, the number of cigarettes smoked per day was significantly higher in hypertensive participants compared to normotensive participants. A study in Albania among young adults showed that age, education, wealth index, religion, physical activity, health insurance, and gender had a significant effect on the prevalence of hypertension [22]. In contrast, a study in Kenya showed that people with a BMI ≥ 25 were 3.05 times more likely to have hypertension; in addition, having a relative with hypertension almost tripled the likelihood of developing hypertension. On the other hand, not drinking alcohol reduces the risk of hypertension by 70% [23]. A study by Thadhani and colleagues also found an association between alcohol consumption and the risk of chronic hypertension in young women aged 25 to 45 years [24].

In young men, increased obesity, high uric acid levels, high resting heart rate, and hypertriglyceridemia were independent factors for hypertension. In young women, the same factors were found, as well as alcohol consumption [25]. In urban areas, people with hypertension were less physically active and more likely to smoke and consume alcohol. Approximately 9.2% of hypertensive people had coexisting diabetes. The mean body weight, BMI, and waist circumference of people with hypertension were significantly higher. In rural areas, a similar association was observed with the exception of alcoholism and diabetes [21].

Psychosocial factors may also contribute to the increased prevalence of hypertension in the younger population [26]. Research suggests that young adults with elevated blood pressure may have a slightly increased risk of cardiovascular events later in life [27].

The most common lipid abnormality in the entire study population was elevated levels of LDL. Elevated TC was observed in 22.6%, low HDL in 13.9%, and elevated TG in 7.8% of the study population. There were no significant differences in the prevalence of lipid disorders between men and women. Studies show that one in ten Balearic adolescents has at least one abnormal lipid concentration. The overall prevalence of dyslipidemia was 13.7% (boys 14.9%; girls 12.9%) [28]. A study involving Mexican adolescents showed that one in two adolescents had at least one abnormal lipid level. The most common dyslipidemia was low HDL-chol levels. Body mass index and abdominal obesity were associated with at least one abnormal lipid level [29]. Cholesterol synthesis markers are higher in adolescents and young adults with type 2 diabetes than in lean subjects and are positively correlated with BMI, TG, hyperinsulinemia, hyperglycemia, and inflammation [30].

TC, LDL, and TG levels were significantly higher (at 25.2, 36.5, and 32.8 mg/dL, respectively) in participants with hypertension than in participants with normal blood pressure. HDL levels were significantly lower in hypertensive participants (56.9 vs. 64.6 mg/dL). The study by Chruściel et al. indicated that elevated systolic blood pressure significantly correlates with HDL cholesterol levels among young adults [12]. A study of a population from Bangladesh showed that serum levels of TC, TG, and LDL were higher, while HDL levels were lower, in hypertensive individuals compared to those with normal blood pressure, which was statistically significant [20]. This is confirmed by the study by Chen and Cheng, which showed that in the lipid profiles, total cholesterol, low-density lipoprotein cholesterol (LDL-c), and non-HDL-c were higher in the hypertensive population (*p* < 0.001) [31].

Participants with abnormal TC levels had an almost six-fold higher risk of hypertension than those with acceptable TC levels (OR = 5.89). Elevated LDL and TG levels contributed to an increased risk of hypertension (OR = 5.38 and 9.75, respectively). The study by Xi et al. confirms that being male, living in urban areas, smoking, and being obese, as well as central obesity, hypertension, and diabetes, are positively correlated with dyslipidemia; alcohol consumption was associated with a lower risk of dyslipidemia [32]. Diabetes, higher BMI, reduced levels of physical activity, and increased waist circumference also contributed significantly to the risk of hypertension [21]. Elevated systolic, diastolic, and LDL blood pressures of young US adults were associated with an increased risk of cardiovascular disease in later life [11]. Men were more likely to be hypertensive (OR = 1.23), and those with hyperglycemia had a 2.83 times higher risk of hypertension. The chance of hypertension increased significantly with the severity of obesity [33].

Hypercholesterolemia is an asymptomatic disorder that occurs years before the onset of myocardial infarction, stroke, or sudden cardiovascular death. Detection of hypercholesterolemia in young adulthood is one of several important steps, along with smoking cessation, blood pressure control, diet education, and exercise promotion, that can be taken in the primary prevention of cardiovascular disease [34]. According to the study by Sesso et al., the baseline lipid levels, especially HDL-C and non-HDL-C, and the TC/HDL-C ratio are associated with an increased risk of hypertension [9]. In the prevention and control of hypertension, attention should be paid to the control of lipid metabolism [35].

A limitation of our study is that the small study group does not consider the effects of variation in the lipid profile due to diet, physical activity, or medication.

## 5. Conclusions

The results of this study demonstrated that participants with hypertension are more likely than normotensive subjects to have elevated levels of TC, LDL, and TG. The prevalence of hypertension is significantly higher in young men than in women. BMI was associated with the prevalence of hypertension and elevated TC levels. Spending more than 2 h a day in front of a computer contributed to the prevalence of hypertension and elevated TG levels. Participants with hypertension smoked a higher number of cigarettes daily compared to those with normotension.

These results could help develop future strategies to prevent hypertension and dyslipidemia through proper lifestyle changes or medical management or by combining both. Hypertensive patients should measure their BP and lipid profiles at regular intervals throughout their primary care.

## Figures and Tables

**Table 1:** Characteristics of the study population.

Variable	Women (*n* = 89)	Men (*n* = 26)	*p*
Age *	20.9 (1.2)	21.2 (1.5)	0.282
Height (cm) *	165.2 (5.9)	177.4 (8.1)	**<0.001**
Weight (kg) *	57.0 (8.5)	73.0 (11.0)	**<0.001**
BMI (kg/m^2^) *	20.9 (2.7)	23.1 (2.3)	**<0.001**
Body fat percentage *	24.1 (5.2)	17.0 (6.0)	**<0.001**
SBP (mm Hg) *	117.8 (11.7)	134.3 (14.5)	**<0.001**
DBP(mm Hg) *	71.3 (6.9)	72.92(10.1)	0.345
TC [mg/dL] *	160.8 (28.8)	172.6 (36.2)	0.087
HDL [mg/dL] *	62.1 (14.5)	57.7 (12.9)	0.169
LDL [mg/dL] *	103.8 (37.5)	109.8 (38.9)	0.411
TG [mg/dL] *	76.26 (36.7)	85.9 (44.5)	0.343
Place of residence ^†^			
City > 100,000 inhabitants	26 (29.2)	11 (42.3)	0.082
City < 100,000 inhabitants	17 (19.1)	8 (30.8)	
Village	46 (51.7)	7 (26.9)	
Weekday time spent in front of the TV ^†^			
No	39 (43.8)	12 (46.2)	0.872
Up to 2 h	40 (44.9)	12 (46.2)	
Over 2 h	10 (11.2)	2 (7.7)	
Weekend time spent in front of the TV ^†^			
No	17 (19.1)	6 (23.1)	0.898
Up to 2 h	55 (61.8)	15 (57.7)	
Over 2 h	17 (19.1)	5 (19.2)	
Weekday time spent sitting in front of the computer ^†^			
No	11 (12.4)	1 (3.8)	0.154
Up to 2 h	41 (46.1)	9 (34.6)	
Over 2 h	37 (41.6)	16 (61.5)	
Weekend time spent sitting in front of the computer ^†^			
No	10 (11.2)	1 (3.8)	**0.003**
Up to 2 h	41 (46.1)	4 (15.4)	
Over 2 h	38 (42.7)	21 (80.8)	
Tobacco use ^†^			
Yes	23 (25.8)	9 (34.6)	0.379
No	66 (74.2)	17 (65.4)	
Duration of smoking (months) *	43.78 (23.02)	40.67 (29.82)	0.754
Number of cigarettes smoked daily *	6.87 (7.44)	6.00 (6.54)	0.761

*—Mean (SD); ^†^—*n* (%); BMI—body mass index; DBP—diastolic blood pressure; HDL—high-density lipoprotein; LDL—low-density lipoprotein; SBP—systolic blood pressure; TC—total cholesterol; TG—triglyceride. Significant associations are highlighted in bold.

**Table 2:** Prevalence of blood pressure status according to sex.

Blood Pressure Status	Total Sample	Women	Men	*p*
Normal	50 (43.5)	47 (52.8)	3 (11.5)	**<0.001**
Elevated	30 (26.0)	23 (25.8)	7 (26.9)	
Hypertension	35 (30.5)	19 (21.3)	16 (61.5)	

Data presented as *n* (%). Significant associations are highlighted in bold.

**Table 3:** Variables influencing the prevalence of hypertension.

Variable	Blood Pressure Status			
	Normal	Elevated	Hypertension	*p*
Age *	20.9 (1.2)	20.7 (1.1)	21.3 (1.4)	0.222
Weight (kg) *	55.0 (7.3)	61.7 (8.9)	67.7 (13.6)	**<0.001**
BMI (kg/m^2^) *	20.0 (2.3)	22.1 (2.3)	22.7 (3.0)	**<0.001**
Body fat percentage *	21.9 (5.6)	24.0 (6.0)	21.9 (6.9)	0.292
Place of residence ^†^				
City > 100,000 inhabitants	19 (38.0)	3 (10.0)	15 (42.9)	**0.034**
City < 100,000 inhabitants	8 (16.0)	9 (30.0)	8 (22.9)	
Village	23 (46.0)	18 (60.0)	12 (34.3)	
Weekday time spent in front of the TV ^†^				
No	25 (50.0)	10 (33.3)	16 (45.7)	0.472
Up to 2 h	22 (44.0)	16 (53.3)	14 (40.0)	
Over 2 h	3 (6.0)	4 (13.3)	5 (14.3)	
Weekend time spent in front of the TV ^†^				
No	12 (24.0)	5 (16.7)	6 (17.1)	0.659
Up to 2 h	31 (62.0)	17 (56.7)	22 (62.9)	
Over 2 h	7 (14.0)	8 (26.7)	7 (20.0)	
Weekday time spent sitting in front of the computer ^†^				
No	9 (18.0)	2 (6.7)	1 (2.9)	0.067
Up to 2 h	24 (48.0)	13 (43.3)	13 (37.1)	
Over 2 h	17 (34.0)	15 (50.0)	21 (60.0)	
Weekend time spent sitting in front of the computer ^†^				
No	6 (12.0)	4 (13.3)	1 (2.9)	**0.031**
Up to 2 h	26 (52.0)	9 (30.0)	10 (28.6)	
Over 2 h	18 (36.0)	17 (56.7)	24 (68.6)	
Tobacco use ^†^				
Yes	10 (20.0)	11 (36.7)	11 (31.4)	0.232
No	40 (80.0)	19 (63.3)	24 (68.6)	
Duration of smoking (months) *	42.7 (24.7)	46.4 (25.2)	39.5 (26.0)	0.815
Number of cigarettes smoked daily *	2.5 (1.5)	7.0 (5.5)	10.0 (9.7)	**0.047**

*—Mean (SD); ^†^—*n* (%); BMI—body mass index; DBP—diastolic blood pressure; HDL—high-density lipoprotein; LDL—low-density lipoprotein; SBP—systolic blood pressure; TC—total cholesterol; TG—triglyceride. Significant associations are highlighted in bold.

**Table 4:** Prevalence of abnormal blood lipid values.

Lipids Status		Total Sample	Women	Men	*p*
TC	Acceptable	89 (77.4)	71 (79.8)	18 (69.2)	0.258
	Abnormal	26 (22.6)	18 (20.2)	8 (30.8)	
HDL	Acceptable	99 (86.1)	76 (85.4)	23 (88.5)	0.691
	Abnormal	16 (13.9)	13 (14.6)	3 (11.5)	
LDL	Acceptable	71 (61.7)	57 (64.0)	14 (53.8)	0.346
	Abnormal	44 (38.3)	32 (36.0)	12 (46.2)	
TG	Acceptable	106 (92.2)	84 (94.4)	22 (84.6)	0.224
	Abnormal	9 (7.8)	5 (5.6)	4 (15.4)	

Data presented as *n* (%). HDL—high-density lipoprotein; LDL—low-density lipoprotein; TC—total cholesterol; TG—triglyceride.

**Table 5:** Variables that influence the prevalence of abnormalities of TC and HDL.

Variable	Lipids Status					
	TC			HDL		
	Abnormal	Acceptable	*p*	Abnormal	Acceptable	*p*
Age *	21.4 (1.6)	20.9 (1.1)	0.209	21.0 (1.6)	21.0 (1.2)	0.581
BMI (kg/m^2^) *	22.5 (3.3)	21.1 (2.5)	**0.025**	21.5 (2.6)	21.4 (2.8)	0.815
Body fat percentage *	23.3 (6.9)	22.2 (5.9)	0.414	23.7 (5.5)	22.3 (6.2)	0.390
Place of residence ^†^						
City > 100,000 inhabitants	7 (26.9)	30 (33.7)	0.704	3 (18.8)	34 (34.3)	0.332
City < 100,000 inhabitants	7 (26.9)	18 (20.2)		3 (18.8)	22 (22.2)	
Village	12 (46.2)	41 (46.1)		10 (62.5)	43 (43.4)	
Weekday time spent in front of the TV ^†^						
No	9 (34.6)	42 (47.2)	0.519	7 (43.8)	44 (44.4)	0.478
Up to 2 h	14 (53.8)	38 (42.7)		6 (37.5)	46 (46.5)	
Over 2 h	3 (11.5)	9 (10.1)		3 (18.8)	9 (9.1)	
Weekend time spent in front of the TV ^†^						
No	2 (7.7)	21 (23.6)	0.155	2 (12.5)	21 (21.2)	0.365
Up to 2 h	17 (65.4)	53 (59.6)		9 (56.3)	61 (61.6)	
Over 2 h	7 (26.9)	15 (16.9)		5 (31.3)	17 (17.2)	
Weekday time spent sitting in front of the computer ^†^						
No	1 (3.8)	11 (12.4)	0.457	1 (6.3)	11 (11.1)	0.361
Up to 2 h	12 (46.2)	38 (42.7)		5 (31.3)	45 (45.5)	
Over 2 h	13 (50.0)	40 (44.9)		10 (62.5)	43 (43.4)	
Weekend time spent sitting in front of the computer ^†^						
No	3 (11.5)	8 (9.0)	0.605	1 (6.3)	10 (10.1)	0.854
Up to 2 h	8 (30.8)	37 (41.6)		6 (37.5)	39 (39.4)	
Over 2 h	15 (57.7)	44 (49.4)		9 (56.3)	50 (50.5)	
Tobacco use ^†^						
Yes	8 (30.8)	24 (27.0)	0.703	6 (37.5)	26 (26.3)	0.352
No	18 (69.2)	65 (73.0)		10 (62.5)	73 (73.7)	
Duration of smoking (months) *	47.7 (30.4)	41.3 (23.0)	0.530	45.3 (20.8)	42.3 (25.8)	0.794
Number of cigarettes smoked daily *	6.0 (9,3)	6.8 (6.4)	0.779	4,8 (6.6)	7.04 (7.3)	0.502

*—Mean (SD); ^†^—*n* (%); BMI—body mass index; DBP—diastolic blood pressure; HDL—high-density lipoprotein; LDL—low-density lipoprotein; SBP—systolic blood pressure; TC—total cholesterol; TG—triglyceride. Significant associations are highlighted in bold.

**Table 6:** Variables that influence the prevalence of abnormalities of LDL and TG.

Variable	Lipids Status					
	LDL			TG		
	Abnormal	Acceptable	*p*	Abnormal	Acceptable	*p*
Age *	21.2 (1.4)	20.8 (1.1)	0.189	21.6 (1.9)	20.9 (1.2)	0.396
BMI (kg/m^2^) *	21.7 (3.1)	21.2 (2.5)	0.298	23.0 (3.2)	21.2 (2.7)	0.063
Body fat percentage *	22.4 (6.6)	22.5 (5.9)	0.936	23.5 (5.2)	22.4 (6.2)	0.623
Place of residence ^†^						
City > 100,000 inhabitants	12 (27.3)	25 (35.2)	0.670	1 (11.1)	36 (34.0)	0.159
City < 100,000 inhabitants	10 (22.7)	15 (21.1)		4 (44.4)	21 (19.8)	
Village	22 (50.0)	31 (43.7)		4 (44.4)	49 (46.2)	
Weekday time spent in front of the TV ^†^						
No	17 (38.6)	34 (47.9)	0.516	4 (44.4)	47 (44.3)	0.448
Up to 2 h	21 (47.7)	31 (43.7)		3 (33.3)	49 (46.2)	
Over 2 h	6 (13.6)	6 (8.5)		2 (22.2)	10 (9.4)	
Weekend time spent in front of the TV ^†^						
No	7 (15.9)	16 (22.5)	0.383	1 (11.1)	22 (20.8)	0.482
Up to 2 h	26 (59.1)	44 (62.0)		5 (55.6)	65 (61.3)	
Over 2 h	11 (25.0)	11 (15.5)		3 (33.3)	19 (17.9)	
Weekday time spent sitting in front of the computer ^†^						
No	1 (2.3)	11 (15.5)	0.079	0 (0.0)	12 (11.3)	**0.027**
Up to 2 h	21 (47.7)	29 (40.8)		1 (11.1)	49 (46.2)	
Over 2 h	22 (50.0)	31 (43.7)		8 (88.9)	45 (42.5)	
Weekend time spent sitting in front of the computer ^†^						
No	4 (9.1)	7 (9.9)	0.400	0 (0.0)	11 (10.4)	0.061
Up to 2 h	14 (31.8)	31 (43.7)		1 (11.1)	44 (41.5)	
Over 2 h	26 (59.1)	33 (46.5)		8 (88.9)	51 (48.1)	
Tobacco use ^†^						
Yes	14 (31.8)	18 (25.4)	0.452	4 (44.4)	28 (26.4)	0.247
No	30 (68.2)	53 (74.6)		5 (55.6)	78 (73.6)	
Duration of smoking (months) *	46.1 (25.7)	40.4 (24.2)	0.531	43.0 (26.8)	42.9 (24.7)	0.994
Number of cigarettes smoked daily *	6.6 (8.5)	6.7 (6.1)	0.971	4.5 (7.7)	6.9 (7.1)	0.531

*—Mean (SD); ^†^—*n* (%); BMI—body mass index; DBP—diastolic blood pressure; HDL—high-density lipoprotein; LDL—low-density lipoprotein; SBP—systolic blood pressure; TC—total cholesterol; TG—triglyceride. Significant associations are highlighted in bold.

**Table 7:** Associations between lipid level and blood pressure.

Lipids	Blood Pressure Status			
	Normal	Elevated	Hypertension	*p*
TC [mg/dL]	150.7 (24.5)	170.2 (34.0)	175.9 (30.2)	**<0.001**
HDL [mg/dL	64.6 (12.1)	60.4 (15.7)	56.9 (14.8)	**0.046**
LDL [mg/dL]	89.7 (31.4)	106.5 (35.0)	126.2 (38.6)	**<0.001**
TG [mg/dL]	65.7 (24.4)	76.3 (36.5)	98.5 (48.6)	**0.003**

Data presented as mean (SD); HDL—high-density lipoprotein; LDL—low-density lipoprotein; TC—total cholesterol; TG—triglyceride. Significant associations are highlighted in bold.

**Table 8:** Odds ratios for the prevalence of hypertension depending on the state of blood lipids.

Blood Pressure Status	Lipids Status			
	TC			
	Acceptable	Abnormal		*p*
	*n* (%)	*n* (%)	OR (95% CI)	
Normal	48 (53.9)	2 (7.7)	0.07 (0.02–0.32)	**<0.001**
Elevated	22 (24.7)	8 (30.8)	1.35 (0.52–3.54)	0.537
Hypertension	19 (21.3)	16 (61.5)	5.89 (2.31–15.07)	**<0.001**
HDL				
	Acceptable	Abnormal		*p*
	*n* (%)	*n* (%)	OR (95% CI)	
Normal	48 (48.5)	2 (12.5)	0.15 (0.03–0.70)	**0.016**
Elevated	23 (23.2)	7 (43.8)	2.57 (0.86–7.66)	0.090
Hypertension	28 (28.3)	7 (43.8)	1.97 (0.67–5.81)	0.218
LDL				
	Acceptable	Abnormal		*p*
	*n* (%)	*n* (%)	OR (95% CI)	
Normal	42 (59.2)	8 (18.2)	0.15 (0.06–0.38)	**<0.001**
Elevated	17 (23.9)	13 (29.5)	1.33 (0.57–3.11)	0.5067
Hypertension	12 (16.9)	23 (52.3)	5.38 (2.28–12.69)	**<0.001**
TG				
	Acceptable	Abnormal		*p*
	*n* (%)	*n* (%)	OR (95% CI)	
Normal	50 (47.2)	0 (0.0)	16.98 (0.96–299.24)	0.0530
Elevated	28 (26.4)	2 (22.2)	0.80 (0.16–4.06)	0.7837
Hypertension	28 (26.4)	7 (77.8)	9.75 (1.91–49.75)	**0.006**

HDL—high-density lipoprotein; LDL—low-density lipoprotein; TC—total cholesterol; TG—triglyceride. Significant associations are highlighted in bold.

## Data Availability

The data presented in this study are not publicly available due to confidentiality reasons. These data are available on request from the corresponding author.

## Supplementary Materials

The following supporting information can be downloaded at: https://www.mdpi.com/article/10.3390/ijerph20020982/s1, Table S1. Post hoc analysis of the prevalence of blood pressure categories and place of residence.
