# Supplementary material for: Association and Risk Factors for Hypertension and Dyslipidemia in Young Adults from Poland
Source: Int J Environ Res Public Health. 2023 Jan 5;20(2):982. doi: 10.3390/ijerph20020982 (PMC9858900; doi:10.3390/ijerph20020982)
Supplement: Supplementary file 1 [file ijerph-20-00982-s001.zip › ijerph-2065204-supplementary.pdf]

Table S1. Post hoc analysis of the prevalence of blood pressure categories and place of residence.

Variables			Place of Residence			Total
			**City < 100,000 Inhabitants**	**City >100,000 Inhabitants**	**Village**	
Blood pressure status	Normal	n	8_a	19_a	23_a	50
		%	32.0	51.4	43.4	43.5
	Elevated	n	**9_a**	**3_b**	**18_a**	30
		%	36.0	8.1	34.0	26.1
	Hypertension	n	8_a	15_a	12_a	35
		%	32.0	40.5	22.6	30.4
Total		n	25	37	53	115
		%	100.0	100.0	100.0	100.0

post hoc analysis - column proportions with Bonferroni correction, significant associations are highlighted in bold.
